# Design and Experiment of an Automatic Leveling System for Tractor-Mounted Implements

**DOI:** 10.3390/s25123707

**Published:** 2025-06-13

**Authors:** Haibin Yao, Engen Zhang, Yufei Liu, Juan Du, Xiang Yin

**Affiliations:** 1 School of Agricultural Engineering and Food Science, Shandong University of Technology, Zibo 255000, China; yao18734274583@163.com (H.Y.); zeg_nice2mu@163.com (E.Z.); dujuan@sdut.edu.cn (J.D.); 2 College of Biosystems Engineering and Food Science, Zhejiang University, Hangzhou 310058, China; yufeiliu@zju.edu.cn

**Keywords:** tractor, tractor-mounted implement, roll angle, automatic leveling, dual closed-loop fuzzy PID

## Abstract

The body roll of the tractor propagates through its rigid hitch system to the mounted implement, causing asymmetrical soil penetration depths between the implement’s lateral working elements, which affects the operational effectiveness of the implement. To address this issue, this study developed an automatic leveling system based on a dual closed-loop fuzzy Proportional-Integral-Derivative (PID) algorithm for tractor-mounted implements. The system employed an attitude angle sensor to detect implement posture in real time and utilized two double-acting hydraulic cylinders to provide a compensating torque for the implement that is opposite to the direction of the body’s roll. The relationship model between the implement’s roll angle and the actuator’s response time was established. The controller performed implement leveling by regulating the spool position and holding time of the solenoid directional valve. Simulink simulations showed that under the control of the dual closed-loop fuzzy PID algorithm, the implement’s roll angle adjusted from 10° to 0° in 1.72 s, which was 56.89% shorter than the time required by the fuzzy PID algorithm, with almost no overshoot. This demonstrates that the dual closed-loop fuzzy PID algorithm outperforms the traditional fuzzy PID algorithm. Static tests showed the system adjusted the implement roll angle from ±10° to 0° within 1.3 s. Field experiments demonstrated that the automatic leveling system achieved a maximum absolute error (*MaxAE*) of 0.91°, a mean absolute error (*MAE*) of 0.19°, and a root mean square error (*RMSE*) of 0.28°, with errors within 0.5° for 92.52% of the time. Results from terrain mutation tests indicate that under a sudden 5° vehicle roll angle change, the system confines implement deviation to ±1.5°. The system exhibits high control precision, stability, and robustness, fulfilling the demands of tractor-mounted implement leveling.

## 1. Introduction

As the primary power source in agricultural production, tractors commonly use a three-point hitch to connect various implements for plowing, planting, management, and harvesting [1,2]. During field operations, uneven terrain induces lateral roll motion of the tractor body, which propagates through the rigid hitch system to the mounted implement. This results in inconsistent soil penetration depths between implement’s lateral working elements, thereby degrading operational performance [3,4,5]. For example, when a tractor-mounted rotary tiller operates on uneven terrain, causing tiller rolls coupled with tractor’s tilt, consequently degrading post-tillage soil surface uniformity. Under extreme conditions, this phenomenon may disrupt the hardpan layer’s structural integrity and induce tiller blade fracture [6].

Regarding automatic leveling of tractor-mounted implements, numerous studies have been conducted. For instance, Fan et al. [7] designed an electro-hydraulic hitch profiling control system based on a Proportional-Integral-Derivative (PID) algorithm with deadband compensation. This system mitigates implement roll motion by integrating a hydraulically driven rotary device between the tractor’s three-point hitch and the implement. Shao et al. [8] designed a lateral attitude adjustment system for mounted implements in hilly terrain based on a fuzzy PID algorithm. This system employed two double-acting hydraulic cylinders as the adjustment mechanism. Under the control of two solenoid proportional valves, the hydraulic cylinders operate synchronously to regulate the implement’s roll attitude. This design ensures that the implement meets the operational requirements for hilly terrain. Zhou et al. [6] proposed a leveling structure for rotary tillers, implementing closed-loop control of hydraulic leveling cylinders to regulate lateral tilt. This approach significantly improved soil surface uniformity and tillage depth consistency. Gürkan et al. [9] developed a laser-guided land-leveling machine that utilizes bilateral hydraulic cylinders to achieve fixed-axis rotation relative to the implement. This system achieves a leveling accuracy of up to 0.05° on hard ground. Xin et al. [10] introduced a genetic algorithm optimized rotary actuator interfaced between the tractor’s three-point hitch and the implement, demonstrating adaptability to sloped field conditions with gradients exceeding 20° Chukewad et al. [11] proposed a leveling scheme for tractor-mounted implements by integrating two control strategies: a position-velocity cascaded PID algorithm for individual hydraulic cylinders and a rolling cooperative control algorithm for dual cylinders. This scheme synchronizes the actuation states of two independent hydraulic cylinders to drive the tractor’s lower links, thereby achieving precise implement leveling. Yu et al. [12] proposed a rolling angle estimation algorithm for tractor-mounted implements based on a four-bar linkage mathematical model and a Kalman filter, and designed an implement leveling system using a hydraulic cylinder installed at the left lift link position of the tractor’s original three-point hitch system as the actuator. The system achieves implement leveling by extending and retracting the hydraulic cylinder piston rod to drive the left lower link.

Overall, two dominant technical approaches exist in the field of research on automatic leveling actuators for implements. The first involves installing electro-hydraulic rotary actuators between the tractor’s three-point hitch and the implement to achieve leveling [6,7,10]. However, control systems based on this design exhibit limited universality, struggling to adapt to multiple implement types. The second approach retrofits the tractor hitch mechanism by replacing the original left and right lifting links with hydraulic cylinders equipped with piston-rod stroke sensors. During operation, the extension/retraction strokes of each cylinder are independently controlled based on roll angle deviation between the implement and the tractor, thereby achieving precise leveling [8,11]. While this system offers stronger adaptability, it is challenged by complex control parameter tuning and higher overall costs.

Regarding leveling control algorithms, existing studies primarily employ classical PID control or single fuzzy PID control [13,14]. The implement’s automatic leveling system faces multiple time-varying disturbances, including periodic tractor vibrations, load shocks from terrain variations, and random lateral oscillations [15,16]. The classical PID algorithm is only suitable for simple, linear, parameter-stable systems. Under disturbances, it generates significant control errors [17]. The conventional fuzzy PID algorithm, despite dynamically adjusting PID parameters via fuzzy logic, operates within a single feedback loop. This makes it better suited for scenarios of moderately complex scenarios [18,19,20]. For implement leveling systems, which are highly non-linear and subject to numerous disturbances, both algorithms exhibit subpar performance.

To address the leveling control requirements for tractor-mounted implements while balancing control accuracy and response speed, this study proposes a reverse compensation strategy for the implement roll attitude. Two double-acting single-piston hydraulic cylinders with opposing motion sequences served as actuators, where one cylinder extended its piston rod while the other retracted, generating a compensating torque opposing the tractor body’s roll direction to achieve implement leveling. These cylinders, driven by a single solenoid directional valve, are installed at the original mounting positions of the tractor’s left and right lifting links. Based on this strategy, a mathematical model linking the implement roll angle to the actuator response time was established. An automatic leveling system for tractor-mounted implements utilizing a dual closed-loop fuzzy PID control algorithm was developed. In this system, an attitude sensor monitors the implement roll angle in real time, while the controller dynamically adjusts the solenoid directional valve’s spool position and dwell time based on the deviation between target and measured roll angles. Simulink simulations demonstrated that the dual closed-loop fuzzy PID approach outperforms conventional fuzzy PID in implement’s automatic leveling control. Static tests and field trials demonstrated that the proposed system achieved high precision and robust stability, effectively meeting the leveling requirements for tractor-mounted implements operating in uneven terrain.

## 2. Materials and Methods

### 2.1. Implement Leveling System

#### 2.1.1. System Overall Architecture

**Figure 1:**
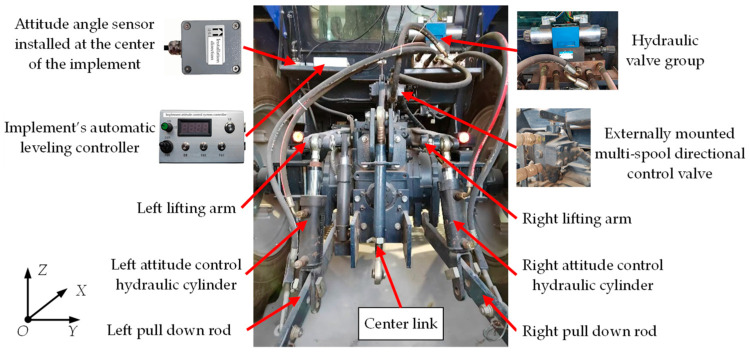
Schematic diagram of tractor rear hitch with implement’s automatic leveling system.

The tractor-mounted implement’s automatic leveling system was developed based on the T954 model tractor produced by Dongfeng Iseki Agricultural Machinery Co., Ltd., (located in Xiangyang, Hubei Province, China). The system was retrofitted from the tractor’s original three-point rear hitch. After modification, the tractor’s rear hitch structure is shown in Figure 1.

**Figure 2:**
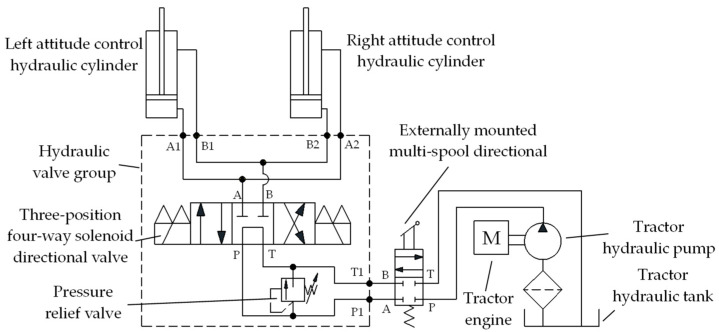
Schematic diagram of the hydraulic control system for the implement leveling actuator.

The implement’s automatic leveling system mainly consisted of a roll attitude adjustment actuator system, an attitude angle sensor, and a leveling controller. The implement roll attitude adjustment actuators were two double-acting single-piston hydraulic cylinders with opposite actions. The attitude angle sensor used was the JY61P six-axis attitude angle sensor produced by WitMotion Shenzhen Co, Ltd., located in Shenzhen, Guangdong Province, China, installed along the longitudinal centerline of the implement to continuously detect its roll attitude. The hydraulic control system schematic diagram of the implement leveling actuator is shown in Figure 2. The system primarily comprises an M-type three-position, four-way solenoid directional valve, a pressure relief valve, and associated components. It was connected to the tractor’s external multi-way valve via pipelines and integrated into the vehicle’s hydraulic system. Detailed models and technical specifications of the hydraulic system components are provided in Table 1. The implement’s automatic leveling controller, based on a PIC18F258 microcontroller produced by Microchip Technology Inc. located in Chandler, Arizona, USA, communicated with the attitude angle sensor through a serial interface to acquire attitude data. It executed the control program to send signals to the solenoid directional valve, adjusting the spool position and maintaining the required time.

#### 2.1.2. System Working Overview

**Figure 3:**
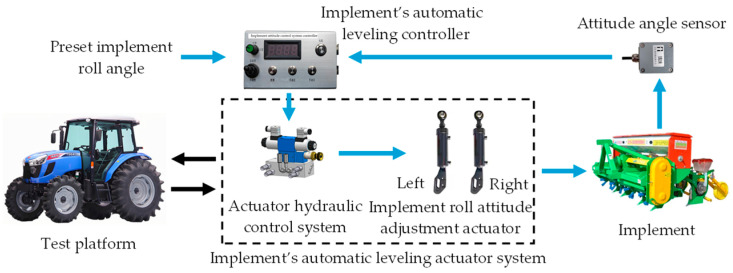
Flowchart of the tractor-mounted implement’s automatic leveling system.

The working principle of the automatic leveling system for the tractor-mounted implement is illustrated in Figure 3. 

During operation, the test platform provided hydraulic power to the implement’s automatic leveling system. When the test platform tilted right, the controller read the preset roll angle and received the real-time roll angle measured by the attitude angle sensor. By comparing these, it determined whether leveling was needed. If so, the control program activated the hydraulic system to extend the left cylinder piston rod and retract the right rod, generating a counterclockwise torque to tilt the implement leftward. Similarly, when the platform tilted left, the controller retracted the left rod and extended the right rod, producing a clockwise torque to tilt the implement rightward.

### 2.2. Design of Implement’s Automatic Leveling Actuator

#### 2.2.1. Kinematic Analysis of Actuator

**Figure 4:**
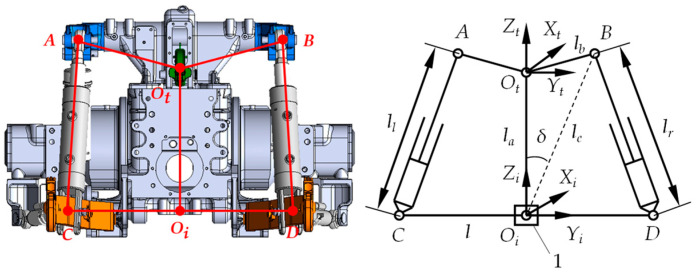
Horizontal alignment of implement relative to tractor.

In the tractor’s coordinate system $O_{t}X_{t}Y_{t}Z_{t}$, the positive $X_{t}$-direction aligned with the tractor’s forward motion. $O_{t}$ marked the articulation point between the center link and tractor body. Points *A* and *B* were the pivot points for the rear hitch’s left/right lift arms and the left/right roll attitude control cylinder piston rods. In the implement’s coordinate system $O_{i}X_{i}Y_{i}Z_{i}$, the positive $X_{i}$-direction aligned with the tractor’s forward motion, with $O_{i}$ at the implement’s center. The attitude sensor was located at $O_{i}$. Points *C* and *D* represented the rear cap ear joints of the left/right roll attitude control cylinders and the left/right lower link pivot points. The implement was modeled as line segment *CD*. After installing the automatic leveling system, the implement’s motion combined the tractor’s traction and relative roll motion. When the implement was horizontal relative to the tractor, the tractor-implement system’s relative position is shown in Figure 4.

When the implement was in a horizontal position relative to the tractor, the actuating mechanism exhibited the geometric relationship shown in Equation (1):

(1) $$\delta =\arccos\;\frac{l_{a}^{2}+l_{c}^{2}-l_{b}^{2}}{{2\;l}_{a}l_{c}}$$

where δ is the angle between $O_{t}O_{I}$ and $O_{t}B$, in degrees; $l_{a}$ is the length of $O_{t}O_{i}$, in meters; $l_{b}$ is the length of $O_{t}B$, in meters; and $l_{c}$ is the length of $O_{i}B$, in meters.

**Figure 5:**
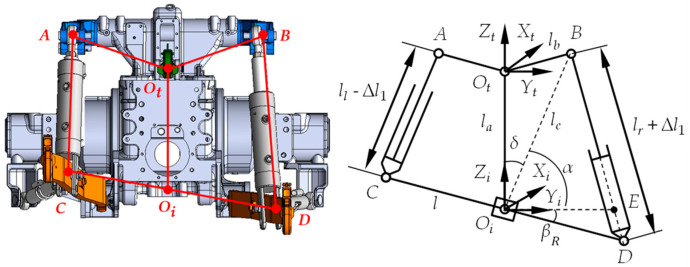
Extreme right tilt position of the implement relative to the tractor.

When the tractor moved along the positive $X_{t}$-axis, neglecting longitudinal tractive motion, the implement’s relative roll motion to the tractor could be approximated as pure rotation in the $O_{i}Y_{i}Z_{i}$ plane [21,22]. The implement right tilt (clockwise rotation) yielded a positive roll angle, while the implement left tilt (counterclockwise rotation) yielded a negative value. Since the left and right roll attitude control cylinder parameters used in this study were identical, and during each adjustment, the extension or retraction travel of both piston rods was minimal, it was assumed that the working stroke of both cylinder piston rod strokes are identical each operation. Therefore, the motion of the implement *CD* in the $O_{i}Y_{i}Z_{i}$ plane could be considered as a rotation around the $X_{i}$-axis, with point $O_{i}$ serving as the center of rotation. Based on the above characteristics of the implement’s motion, a center control method was used to achieve implement roll attitude control. It was assumed that the position of point $O_{i}$ was fixed, and its relative position to the tractor’s $O_{t}$ point remained unchanged. The roll angle β was defined as the angle between *CD* and the $O_{i}X_{i}Y_{i}$ plane [23,24]. Figure 5 shows the maximum right tilt position of the implement, where *CD* rotated clockwise around $O_{i}$ with roll angle $\beta_{R}$.

The functional relationship between the maximum right roll angle $\beta_{R}$ and the piston rods displacement change $\Delta l_{1}$ of the roll attitude control cylinders was given as:

(2) $$\beta_{R}=\arccos\;\frac{l_{c}^{2}+\frac{1}{4}l^{2}-{\left(l_{r}{\;+\;\Delta l}_{1}\right)}^{2}}{l_{c}l}-90{}^{\circ}+\delta$$

where *l* is the defined as the distance between points *C* and *D*, in meters, and $l_{r}$ is the length of the right roll attitude control cylinder *BD* when both the tractor and implement are level, in meters.

**Figure 6:**
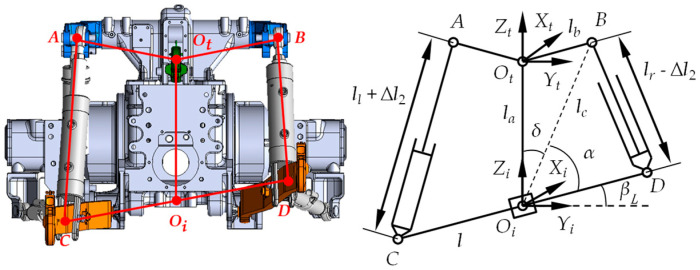
Extreme left tilt position of the implement relative to the tractor.

Figure 6 shows the maximum left tilt position of the implement with counterclockwise rotation and roll angle $\beta_{L}$.

The functional relationship between the maximum left roll angle $\beta_{L}$ and the displacement change $\Delta l_{2}$ of the piston rod in the roll attitude control cylinder was given as:

(3) $$\beta_{L}=90{}^{\circ}-\arccos\;\frac{l_{c}^{2}+\frac{1}{4}l^{2}-{\left(l_{r}-\Delta l_{2}\right)}^{2}}{l_{c}l}-\delta$$

#### 2.2.2. Analyzing the Relationship of Implement Roll Angle and Actuator Response Time

When the implement was tilted to its right limit position, the relationship between the response time *T_P_* of the left roll attitude control hydraulic cylinder piston rod retraction and the retraction distance is given by Equation (4). The relationship between the response time $T_{E}$ of the right roll attitude control hydraulic cylinder piston rod extension and the extension distance is given by Equation (5); both formulations were proposed by Yin et al. [22]:

(4) $$T_{P}=\frac{15\pi ({d_{1}}^{2}-{d_{2}}^{2})\Delta l_{1}}{Q}$$

(5) $$T_{E}=\frac{15\pi {d_{1}}^{2}\;\Delta l_{1}}{Q}$$

where $d_{1}$ is the piston diameter of the attitude-controlled hydraulic cylinder, where $d_{1}=0.08$ m; $d_{2}$ is the piston rod diameter of the attitude-controlled cylinder, where $d_{2}=0.05$ m; and *Q* is the flow rate of the attitude-controlled hydraulic cylinder, in L/min.

While the tractor operated at a constant speed, the hydraulic gear pump’s pressure and flow rate were essentially stable. Thus, it could be approximated that the pressure and flow rate in the hydraulic control system of the attitude adjustment actuator remained stable during operation. To reduce the roll attitude control error caused by the inconsistent extension stroke of the cylinder’s piston rod, the mean quantization method was used to determine the relationship between the machine’s roll angle and the cylinder’s response [25]. Therefore, the formula for calculating the required response time for each 1° rotation of the implement can be expressed as:

(6) $$T_{1}=\frac{T_{E}+T_{P}}{\beta_{S}}$$

It can be approximated that for each 1° rotation of the implement, the solenoid directional valve spool needs to remain at a specific position for a total duration of $T_{1}$.

### 2.3. Design of Implement’s Automatic Leveling Algorithm

#### 2.3.1. Working Principle of Dual Closed-Loop Fuzzy PID

**Figure 7:**
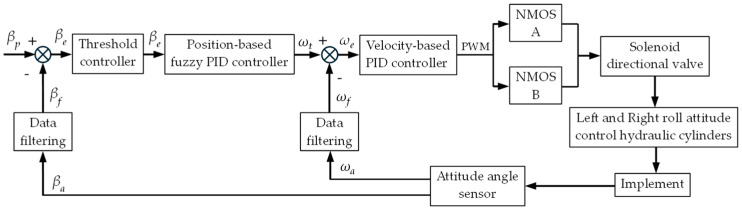
Dual closed-loop fuzzy PID algorithm working principle.

The working principle of the dual closed-loop fuzzy PID algorithm for the tractor-mounted implement’s automatic leveling system is shown in Figure 7.

In Figure 7, $\beta_{a}$ denotes the current roll angle of the implement measured by the attitude angle sensor, $\beta_{f}$ is the filtered roll angle, and $\beta_{p}$ represents the preset desired roll angle. The difference between $\beta_{f}$ and $\beta_{p\;}$, denoted as $\beta_{e\;}$, was inputted into a threshold controller to determine whether attitude adjustment was needed. When adjustment was required, $\beta_{e}$ was inputted into the position-based fuzzy PID controller, which processed it and output the preset roll angular velocity $\omega_{t}$ required to position the implement as desired. The actual roll angular velocity measured by the sensor was $\omega_{a\;}$, and its filtered value was $\omega_{f\;}$. The difference between $\omega_{f}$ and $\omega_{t\;}$, referred to as $\omega_{e\;}$, was inputted into a velocity-type PID controller. After computation and processing, the controller outputted a Pulse-Width Modulation (PWM) signal to control the N-Metal-Oxide-Semiconductor (NMOS) driver module. Through the on-off control of the NMOS driver modules A and B, the spool position and dwell time of the solenoid directional valve were regulated, thereby controlling the extension/retraction of the left/right roll attitude control cylinder piston rods to adjust the implement roll attitude. Finally, the adjusted implement roll attitude from the sensor was fed back to the controller, closing the control loop.

#### 2.3.2. Design of Fuzzy PID Algorithm

The PID algorithm is given by:

(7) $$\;u\left(t\right){=K}_{p}e\left(t\right){+K}_{i}\int_{0}^{t}{e(t)dt+K}_{d}\frac{de(t)}{dt}$$

where u(t) is the controller’s control output; e(t) is the system’s actual error signal; and $K_{p}$, $K_{i}$, $K_{d}$ are the proportional, integral, and derivative gains, respectively.

**Figure 8:**
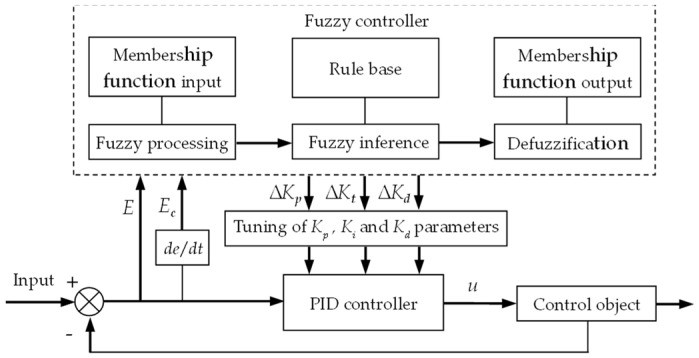
Schematic diagram of the principle of fuzzy PID.

The fuzzy PID control method took the error *E* and the error change rate $E_{c}$ as its inputs. It then applied fuzzy inference rules to adapt the PID parameters in real time according to changes in the system, ultimately generating parameter corrections. The schematic of the fuzzy PID principle is shown in Figure 8.

The fuzzy controller adjusted the three PID parameters $K_{p\;}$, $K_{i}$, and $K_{d}$ according to Equation (8):

(8) $$\left\{\begin{array}{l} K_{p}{\;=K}_{p0}+\Delta K_{p}\\ K_{i}{\;=K}_{i0}+\Delta K_{i}\\ K_{d}{\;=K}_{d0}+\Delta K_{d} \end{array}\right.$$

where $K_{p}$, $K_{I}$, and $K_{d}$ are the PID parameters after fuzzy tuning; $K_{p0}$, $K_{i0}$, and $K_{d0}$ represent the initial values of the PID parameters; and $\Delta K_{p}$, $\Delta K_{I}$, and $\Delta K_{d}$ are the corrections obtained from the fuzzy algorithm.

Define the universal domain of the implement roll angle error *E* as $\left[-18,\;18\right]$, and that of the roll angle error rate $E_{c}$ as [−2.5, 2.5]. The basic domains of the PID tuning coefficients $\Delta K_{p}$, $\Delta K_{i}$, and $\Delta K_{d}$ were defined as [6, 18], [2, 6], and [1.15, 3.45], respectively. To facilitate domain conversion, the fuzzy domain for both *E* and $E_{c}$ were set to $\left[-3,\;3\right]$, and the fuzzy domain for $\Delta K_{p}$, $\Delta K_{i}$, and $\Delta K_{d}$ were set to $\left[-1,\;1\right]$. The quantization factors for the *E* and $E_{c}$ were 0.17 and 1.2, respectively, while the quantization factors for $\Delta K_{p}$, $\Delta K_{i}$, and $\Delta K_{d}$ were 6, 2, and 1.15, respectively. The fuzzy variables *E*, $E_{c}$, $\Delta K_{p}$, $\Delta K_{i}$, and $\Delta K_{d}$ were represented by the set {Negative Big, Negative Medium, Negative Small, Zero, Positive Small, Positive Medium, Positive Big}, respectively expressed as {NB, NM, NS, ZO, PS, PM, PB}. A triangular function with higher precision was selected as the membership function [23,26].

Based on practical operational requirements, the fuzzy self-tuning rules for $K_{p}$, $K_{i}$, and $K_{d}$ in the fuzzy PID algorithm were defined as follows.

(1) When the implement’s roll-angle error *E* was large (PB or NB), $K_{p}$ was increased to accelerate response, while $K_{i}$ was reduced to prevent excessive integral error.

(2) When the error *E* was moderate (NM or PM), a moderate $K_{p}$ was selected to avoid overshoot and oscillation, and a moderate $K_{p}$ was chosen to expedite system stabilization.

(3) When the implement’s roll angle was close to the preset roll angle (error *E* was PS or NS), $K_{p}$ was slightly decreased to maintain the roll angle near the target. At the same time, $K_{i}$ was slightly increased to eliminate steady-state errors more quickly.

(4) If the error *E* was PS or NS and the error rate $E_{c}$ was large (PB or NB), $K_{d}$ was increased to reduce overshoot and enhance stability while avoiding oscillation. If $E_{c}$ was small (PS or NS), then $K_{d}$ was decreased appropriately to maintain system stability.

Based on the prescribed fuzzy PID self-tuning rules, the fuzzy rule sets for $\Delta K_{p}$, $\Delta K_{i}$, and $\Delta K_{d}$ were established, as shown in Table 2.

### 2.4. Implement’s Automatic Leveling Controller Design

#### 2.4.1. Hardware Design

**Figure 9:**
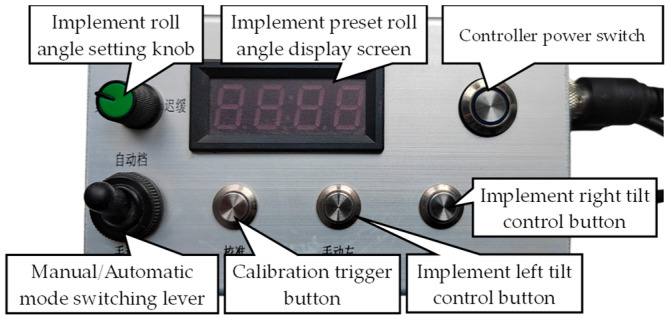
Physical prototype of the tractor-mounted implement’s automatic leveling controller.

The physical prototype of the tractor-mounted implement’s automatic leveling controller is shown in Figure 9, which integrated a power module, main control module, NMOS driver module, and manual operation switch.

**Figure 10:**
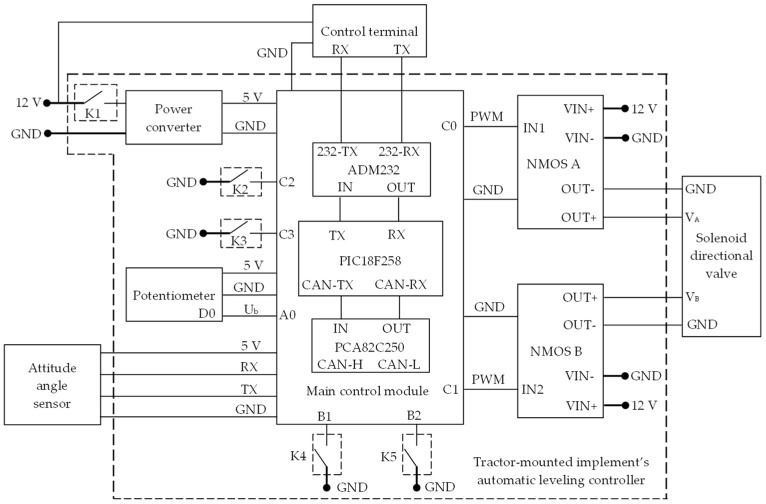
Working principle of implement’s automatic leveling controller. C0, C1, C2, C3, B1, B2, and A0 are digital output pins on the PIC18F258 microcontroller. CANTX is the CAN bus transmit pin of the control core; CANRX is the CAN bus receive pin of the control core. IN1 and IN2 are the trigger inputs for the NMOS driver module; VIN+ and OUT+ are the positive input and output ports of the NMOS driver module, respectively; VIN− and OUT− are the negative input and output ports of the NMOS driver module, respectively. K1 is the power switch; K2 is the calibration switch; K3 is the manual/automatic toggle switch; K4 and K5 are the attitude cylinder control switches.

The working principle of the implement’s automatic leveling controller is shown in Figure 10. The power module, which was a 12 V to 5 V step-down converter controlled by switch K1, converted the 12 V DC power supplied by the tractor into 5 V DC power. The main control module, consisting of a PIC18F258 microcontroller, a PCA82C250 chip produced by NXP Semiconductors located in Eindhoven, the Netherlands, an ADM232 chip produced by Analog Devices Inc. located in Wilmington, Massachusetts, USA, and their peripheral circuits, managed system operations. A JY61P six-axis attitude angle sensor handled data acquisition and transmitted data at 20 Hz via a serial port at 115,200 baud. The two NMOS driver modules, produced by Yueyu Electronics Technology Co., Ltd. located in Shenzhen, Guangdong Province, China, were controlled by PWM pulse signals output from the PIC18F258 microcontroller, which were used to adjust the energizing time of the left and right coils of the solenoid directional valve. A rotary potentiometer was included for setting the preset roll angle, with its output connected to the main control module’s A/D conversion port A0. The controller also had a manual/automatic mode switch K2, an attitude calibration switch K3, and manual control switches K4 and K5 for attitude adjustment. When the switches were closed, the microprocessor detected a low-level signal on the corresponding port, and the microcontroller ran the corresponding preset program.

When the C0 and C1 pins outputted low-level signals, the VIN− and OUT− ports of the NMOS driver modules A and B were disconnected. Consequently, the solenoid directional valve remained inactive, and the left and right roll attitude control cylinders did not move. When the C0 pin outputted a high-level signal and the C1 pin outputted a low-level signal, the VIN− and OUT− ports of NMOS A closed, energizing the coil on the $V_{A}$ side of the solenoid directional valve. This action extended the piston rod of the left cylinder while retracting the rod of the right cylinder, causing the implement to rotate counterclockwise in the roll plane. Conversely, when C0 outputted a low-level signal and C1 outputted a high-level signal, the VIN− and OUT− ports of NMOS B closed, energizing the coil on the $V_{B}$ side of the valve. This retracted the left cylinder’s piston rod and extended the right one, resulting in clockwise rotation of the implement in the roll plane.

#### 2.4.2. Software Design

**Figure 11:**
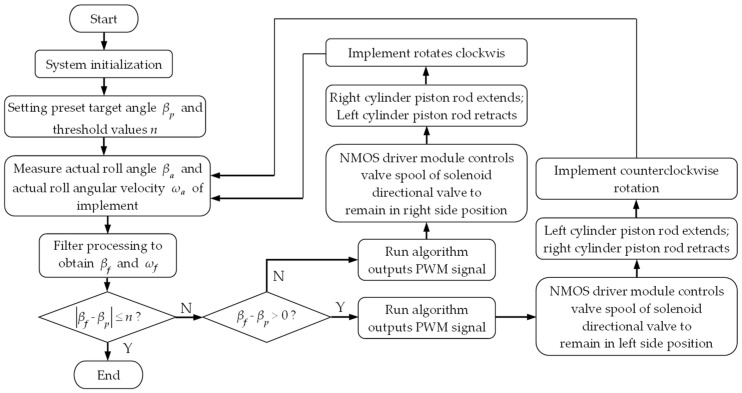
System control flowchart.

The control program for the tractor-mounted implement’s automatic leveling system was developed in C programming language using MPLAB IDE V8.92. The system control flow is shown in Figure 11. Upon system startup, initialization was first performed to calibrate the desired implement roll angle $\beta_{p}$ and the roll angle adjustment threshold *n*. Concurrently, the implement’s actual roll angle $\beta_{a}$ and roll angular velocity $\omega_{a}$ were measured, and filtering processing was applied to these values to obtain the filtered signals $\beta_{f}$ and $\omega_{f\;}$. Based on the absolute error between $\beta_{f}$ and $\beta_{p\;}$, the system determined whether an attitude adjustment was necessary. If a roll attitude adjustment was required, the main control module ran the roll attitude control program. It outputted PWM signals to control NMOS driver modules A and B, which in turn controlled the position and holding time of the solenoid directional valve spool, driving the extension/retraction of the left and right roll attitude control cylinders to adjust the implement’s roll attitude. This process continued until the absolute error between $\beta_{p}$ and $\beta_{f\;}$ was within threshold *n*.

### 2.5. Simulation Analysis of Tractor-Mounted Implement’s Automatic Leveling Control System

#### 2.5.1. Modeling of the Valve-Controlled Hydraulic Cylinder System

The automatic-leveling actuators selected for the implement in this study were double-acting single-piston hydraulic cylinders, controlled by an M-type three-position/four-way solenoid directional valve. This configuration necessitated the development of a mathematical model for a symmetric-valve-controlled asymmetric hydraulic cylinder system [27]. During the derivation of the mathematical model, it was assumed that the control valve was an ideal zero-lapped four-way spool valve, pressure losses in hydraulic pipelines were negligible, and pressure was uniform throughout the same connected region [28]. Under steady-state conditions, the linear flow equation for the cylinder is expressed as:

(9) $$Q_{L}=K_{q}x_{v}-K_{c}p_{L}$$

where $Q_{L}$ is the load flow rate of the hydraulic cylinder, in L/min; $K_{q}$ is the steady-state flow gain of the solenoid valve, in $m^{2}/s$; $x_{v}$ is the spool displacement of the solenoid valve, in meters; $K_{c}$ is the steady-state pressure-flow coefficient of the solenoid valve, in $m^{5}/(\mathrm{Ns})$; and $p_{L}$ is the load pressure of the double-acting single-piston hydraulic cylinder, in Pa.

The flow continuity equation for the hydraulic cylinder is expressed as:

(10) $$Q_{L}=A_{p}\frac{{dx}_{p}}{dt}+\frac{V_{t}}{{2(1+s}^{2}{)\beta }_{e}}\frac{{dp}_{L}}{dt}+{(C}_{\mathrm{ip}}+C_{\mathrm{ep}}{)p}_{L}$$

where $A_{p}$ is the effective area of the hydraulic cylinder, in $m^{2}$; $x_{p}$ is the piston rod displacement, in meters; $V_{t}$ is the cylinder volume, in $m^{3}$; $\beta_{e}$ is the effective bulk modulus; *s* is the area ratio of the non-rod chamber to the rod chamber; and $C_{\mathrm{ip}}$ and $C_{\mathrm{ep}}$ are the internal and external leakage coefficients of the hydraulic cylinder.

Under steady-state conditions, the force balance equation for an asymmetric hydraulic cylinder controlled by a symmetric four-way valve can be expressed as:

(11) $$A_{p}p_{L}=m\frac{d^{2}x_{p}}{{dt}^{2}}+B_{p}\frac{{dx}_{p}}{dt}+{Kx}_{p}+F_{L}$$

where *m* is the total mass of the load, in kg; $B_{p}$ is the viscous damping coefficient, in Ns/m; *K* is the load stiffness, in N/m; *F_L_* is the external load force, N.

Taking the Laplace transform of Equations (9) through (11) and eliminating the intermediate variables $Q_{L}$ and $p_{L}$ yielded the transfer function of the four-way valve-controlled, asymmetric hydraulic cylinder. In actual operation, the system was dominated by inertial load, while the elastic load and viscous damping are relatively small and can be neglected, resulting in the simplified transfer function:

(12) $$H(s)=\frac{\frac{K_{q}}{A_{p}}}{s\left(\frac{s^{2}}{\omega_{h}^{2}}+\frac{{2\xi }_{h}}{\omega_{h}}s+1\right)}$$

(13) $$\omega_{h}=\sqrt{\frac{{2(1+s}^{2}{)\beta }_{e}A^{2}}{{\mathrm{mV}}_{t}}}$$

(14) $$\xi_{h}=\frac{K_{\mathrm{ce}}}{{2A}_{1}}\sqrt{\frac{{2(1+s}^{2}{)\beta }_{e}m}{V_{t}}}$$

where $\omega_{h}$ is the natural frequency of the hydraulic cylinder, in rad/s; $\xi_{h}$ is the cylinder damping ratio; *A* is effective area of the hydraulic cylinder piston, in $m^{2}$; and $K_{\mathrm{ce}}$ is the total flow-pressure coefficient of the cylinder, $K_{\mathrm{ce}}=K_{c}+K_{\mathrm{ip}}+C_{\mathrm{ep}}$.

#### 2.5.2. Simulation Analysis of Implement’s Automatic Leveling System

A simulation model for the tractor-mounted implement’s automatic leveling system was developed in MATLAB/Simulink version 9.12.0.1884302 (R2022a). The parameters are shown in Table 3 [29,30].

To evaluate the performance of the proposed dual closed-loop fuzzy PID algorithm, it was compared with a fuzzy PID algorithm. In the simulation model, the implement’s automatic leveling systems based on both algorithms were established. 

The initial parameters of the PID controller were tuned using the Ziegler–Nichols closed-loop oscillation method. Initially, under pure proportional control mode ($K_{i}$ = 0, $K_{d}$ = 0), the proportional gain $K_{P}$ was gradually increased until the system output exhibited sustained oscillations. The critical gain $K_{c}$ = 12.55 and the critical oscillation period $T_{c}$ = 1.88 s were obtained. The acquired $K_{c}$ and $T_{c}$ values were substituted into Equation (15):

(15) $$\left\{\begin{array}{c} K_{p}=0{.6K}_{c}\\ K_{i}=0.5\frac{K_{c}}{T_{c}}\\ K_{d}=0{.125K}_{c}T_{c} \end{array}\right.$$

According to Equation (15), the calculated initial PID parameters are as follows: $K_{P}$ = 7.53, $K_{i}$ = 3.34, and $K_{d}$ = 2.95. These three parameters were applied to the closed-loop system for step response testing. The initial parameter values were then optimized using the Control System Tuner toolbox in Simulink, resulting in the final determined PID initial parameters: $K_{P}=12$, $K_{i}=4$, and $K_{d}=2.3$.

**Figure 12:**
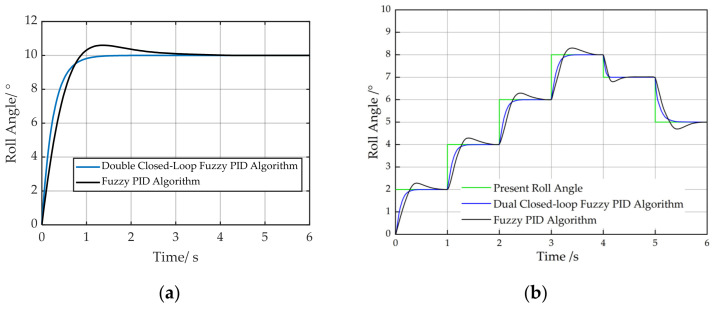
Simulink results. (**a**) Unit step response. (**b**) Step response with disturbance.

Simulations were conducted for both algorithms using identical initial PID parameters. The initial roll angle of the implement was set to 0°, and a 10° step input was applied. The simulation was conducted with a sampling frequency of 20 Hz and a duration of 6 s, and the results are presented in Figure 12a. To validate system performance under complex field conditions, disturbances were introduced in the simulation model, with results shown in Figure 12b.

Under the fuzzy PID algorithm control, the settling time for implement attitude adjustment was 3.99 s, with an overshoot of 0.60°. The dual closed-loop fuzzy PID algorithm achieved a settling time of 1.72 s and exhibited negligible overshoot. Figure 12b demonstrates that the proposed dual closed-loop fuzzy PID controller achieves significant reductions in overshoot and leveling time compared to the conventional fuzzy PID algorithm during disturbed step-response tests. Simulation results demonstrated that the dual closed-loop fuzzy PID algorithm provided superior control performance for the implement’s automatic leveling control compared to the fuzzy PID algorithm.

## 3. Experimental Testing and Analysis

To test the practical performance of the designed tractor-mounted implement’s automatic leveling system, static and field tests were conducted in May 2024 at the Ecological Unmanned Farm of Shandong University of Technology (SDUT). The experimental platform utilized an autonomous tractor developed by SDUT based on the Iseki T954 tractor model, equipped with the 2BMYFC series maize no-tillage fertilization precision planter by Shandong Dahua Machinery Co., Ltd. of Jining, Shandong Province, China. An integrated positioning–orientation receiver independently developed by SDUT was installed on the tractor roof, incorporating a JY61P attitude angle sensor for real-time monitoring the vehicle’s posture. The system performance was evaluated using four indices: maximum absolute error of roll angle (*MaxAE*), mean value $\bar{\beta }$, mean absolute error (*MAE*), and root mean square error (*RMSE*) of the planter’s roll angle, with the calculation formulas provided as:

(16) $$\;\mathrm{MaxAE}=\mathrm{Max}\;\left|\beta_{\mathrm{ai}}-\beta_{p}\right|$$

(17) $$\bar{\beta }=\frac{\sum_{i=1}^{N}\;\beta_{\mathrm{ai}}}{N}$$

(18) $$\mathrm{MAE}=\frac{\sum_{i=1}^{N}\left|\beta_{\mathrm{ai}}-\beta_{p}\right|}{N}$$

(19) $$\mathrm{RMSE}=\sqrt{\frac{\sum_{i=1}^{N}{\left(\beta_{\mathrm{ai}}-{\;\beta }_{p}\right)}^{2}}{N}}\;(i=1,\;2,\;\cdots \;N)$$

The sample size *N* is calculated using Equation (20):

(20) N=f T

where *f* denotes the sampling frequency of the attitude angle sensor (*f* = 20 Hz) and *T* is the total sampling duration, in s.

### 3.1. Static Experiment

#### 3.1.1. Experimental Method and Content

**Figure 13:**
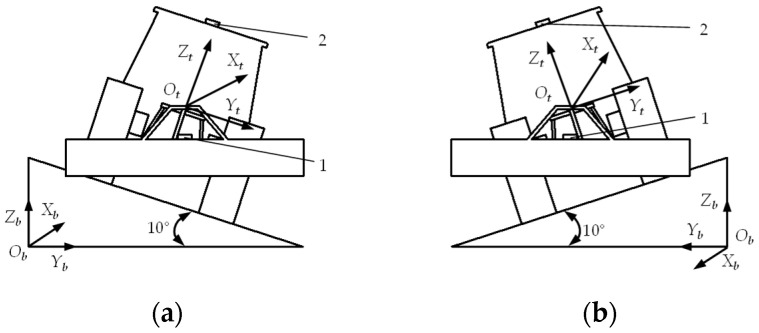
Schematic diagram of static testing. (**a**) Schematic diagram of tractor position in the first phase of testing. (**b**) Schematic diagram of tractor position in the second phase of testing. 1. Attitude angle senso. 2. Positioning–orientation receiver.

The static test bench has a slope of 10° and a length of 6 m, where the implement’s preset angle $\beta_{p}$ and threshold *n* are both 0°. The tractor’s position during the static test is illustrated in Figure 13.

Using a remote control, the driverless tractor was maneuvered onto the test bench such that the positive direction of the $X_{t}$-axis in the tractor’s coordinate system $O_{t}X_{t}Y_{t}Z_{t}$ aligns with the positive $X_{b}$-axis of the test bench coordinate system $O_{b}X_{b}Y_{b}Z_{b}$. After the tractor came to a complete stop, the control program based on the dual closed-loop fuzzy PID algorithm was activated, keeping the tractor stationary for 120 s. Thereafter, the automatic control program was turned off, and the implement roll attitude was manually restored to its pre-adjustment state. Subsequently, the fuzzy PID algorithm control program was restarted, maintaining tractor stillness for another 120 s before shutting down the automatic control to conclude the first phase of testing. For the second phase of testing, the driverless tractor was remotely guided back onto the test bench with the positive $X_{t}$-axis direction now opposite to the test bench’s positive $X_{b}$-axis. The first stage test procedures were then repeated.

#### 3.1.2. Experimental Results and Analysis

Through observation and analysis of the implement roll angle variation curve, it was found that during the initial 0–15 s phase, the roll angle exhibited significant fluctuations due to active leveling operations. In the subsequent 15,120 s phase, however, the implement completed the leveling process, with the roll angle demonstrating negligible fluctuations. Based on the marked disparity in fluctuation characteristics, this study selected the 0–15 s roll angle variation curve as the analytical subject for static test result evaluation. 

**Figure 14:**
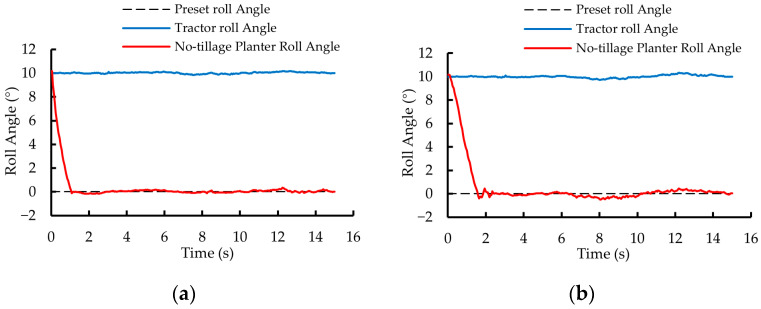
Static test results for the first phase of testing. (**a**) Dual closed-loop fuzzy PID control algorithm. (**b**) Fuzzy PID control algorithm.

In the first phase of testing, the roll angle variation curves of the no-tillage planter controlled by the dual closed-loop fuzzy PID algorithm and the fuzzy PID algorithm are shown in Figure 14a and Figure 14b, respectively. Under the control of the two algorithms, the roll angle of the no-tillage planter was adjusted from 10° to 0° within 1.25 s and 2.45 s, with a *MaxAE* of 0.35° and 0.52°, respectively.

**Figure 15:**
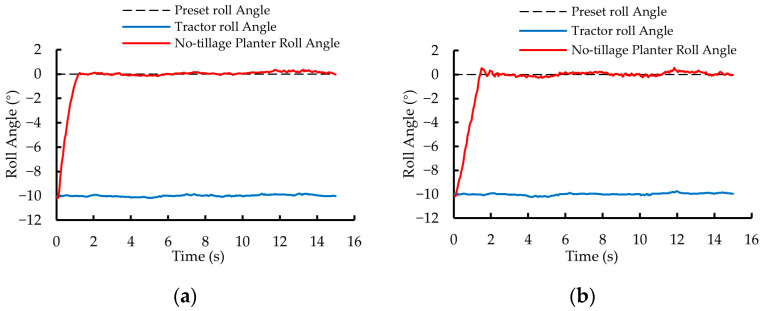
Static test results for the second phase of testing. (**a**) Dual closed-loop fuzzy PID control algorithm. (**b**) Fuzzy PID control algorithm.

In the second phase of testing, the roll angle variation curves of the no-tillage planter under the control of the dual closed-loop fuzzy PID algorithm and the fuzzy PID algorithm are shown in Figure 15a and Figure 15b, respectively. The roll angle of the no-tillage planter was adjusted from −10° to 0° within 1.30 s and 2.50 s, with a *MaxAE* of 0.36° and 0.56°, respectively.

Compared with the fuzzy PID algorithm, the dual closed-loop fuzzy PID algorithm ensures that the no-tillage planter’s leveling process exhibits almost no overshoot, with shorter settling time, smaller roll angle error, and better stability. The results of the static tests demonstrate that the tractor-mounted implement’s automatic leveling system designed in this study achieves high control accuracy and response speed.

### 3.2. Field Experiment

#### 3.2.1. Experimental Method and Content

**Figure 16:**
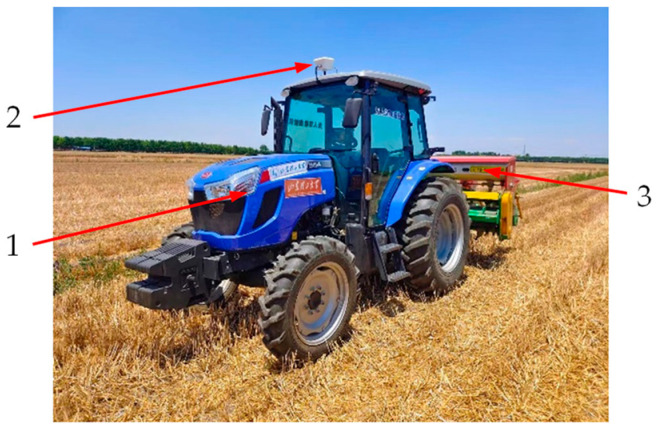
Field experiment scenario and test platform. 1. Unmanned tractor equipped with the implement’s automatic leveling system. 2. Positioning–orientation receiver. 3. Manufactured 2BMYFC series maize residue-cleaning no-tillage planter.

The field experiment scenario and test platform are illustrated in Figure 16.

**Figure 17:**
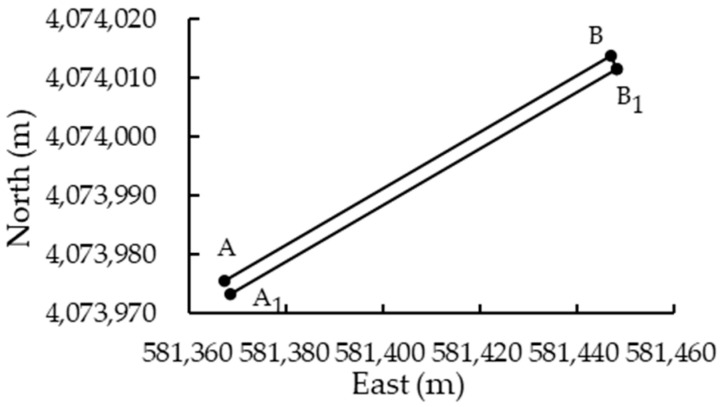
Path planning for field operations of autonomous tractor.

The unmanned farm is located on the North China Plain, with typical ground slope angles ranging from 0.5° to 3.5°. In unmanned driving mode, the tractor-mounted implement’s automatic leveling system, based on the dual closed-loop fuzzy PID algorithm, was activated. The target roll angle of the no-tillage planter was set to 0° with a threshold of 0.5°. The tractor began at point A (581,367.298 m, 4,073,975.609 m), followed the preplanned path (Figure 17), through points B (581,447.087 m, 4,074,013.775 m) and $B_{1}$ (581,448.166 m, 4,074,011.522 m), and terminated at point $A_{1}$ (581,368.377 m, 4,073,973.354 m). Segments AB and $B_{1}A_{1}$ were straight lines. Upon reaching point B, the implement’s automatic leveling system was deactivated, the planter was raised, and the tractor executed a headland turn to $B_{1}$. At $B_{1}$, the planter was lowered, the control system was reactivated, and the tractor proceeded to $A_{1}$, where the system was deactivated again and the planter was retracted. The tractor was then remotely navigated back to its initial position and re-entered unmanned mode. The target roll angle and threshold were kept unchanged at 0° and 0.5°. The fuzzy PID-based control system was restarted, and the tractor repeated the seeding operation along the identical trajectory. Throughout the operation, the roll angles of both the tractor and planter were recorded to evaluate the control performance.

#### 3.2.2. Experimental Results and Analysis

**Figure 18:**
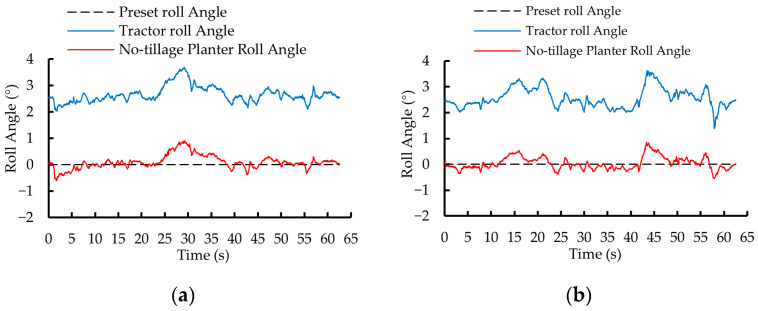
Roll angle variation curves of tractor and no-tillage planter under the dual closed-loop fuzzy PID algorithm control. (**a**) A → B. (**b**) $B_{1}\;\to {\;A}_{1}$.

Figure 18a and Figure 18b show the roll angle variations of the tractor and planter under the dual closed-loop fuzzy PID control algorithm during two predefined path segments: A → B and $B_{1}\;\to {\;A}_{1}$. The data were acquired at a sampling rate of 20 Hz.

**Figure 19:**
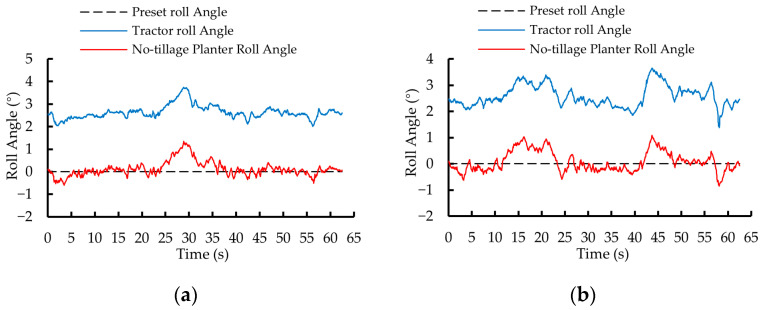
Roll angle variation curves of tractor and no-tillage planter under the fuzzy PID algorithm control. (**a**) A → B. (**b**) $B_{1}\;\to {\;A}_{1}$.

Figure 19a and Figure 19b illustrate the variation in the roll angle of the tractor and planter under the fuzzy PID control algorithm during the two planned path segments: A → B. $B_{1}\to A_{1}$.

The data analysis of the field test results is shown in Table 4.

Compared to the fuzzy PID control algorithm, the dual closed-loop fuzzy PID control algorithm reduces the *MaxAE*, $\bar{\beta }$, *MAE*, and *RMSE* of the roll angle of the no-tillage planter, thereby effectively improving the accuracy and stability of its leveling control. Field tests demonstrate that the automatic leveling system for the tractor-mounted implement, designed in this study, enables automatic roll attitude control and exhibits good response speed, control accuracy, and stability.

### 3.3. System Anti-Interference Capability Verification Under Localized Terrain Irregularities

**Figure 20:**
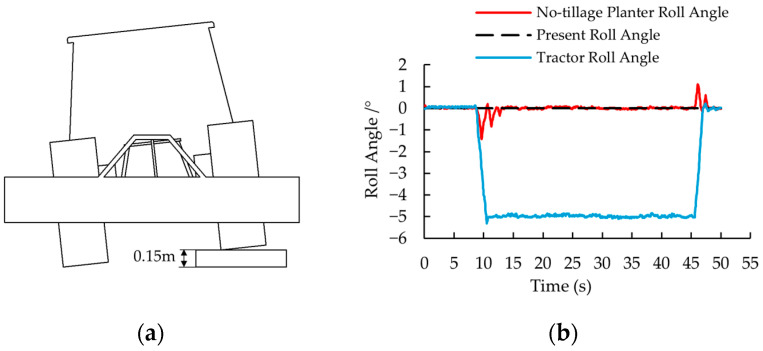
Experimental setup and response under localized terrain irregularity. (**a**) Experimental setup schematic. (**b**) Response results.

Field operating environments are complex, with unpredictable terrain undulations. To evaluate the performance of the designed tractor-mounted implement leveling system when encountering localized terrain irregularities, a dedicated field test was conducted Figure 20a. A step obstacle with a height of 0.15 m and total length of 10 m was constructed. After activating the leveling control system, the tractor was operated at 1 km/h to sequentially pass the right-side front and rear wheels over the step keeping the left wheels grounded. The roll angles of both the implement and tractor body were synchronously recorded to examine the system’s dynamic response performance under sudden elevation changes. Test results are shown in Figure 20b.

As depicted in Figure 20b, when encountering a 0.15 m step obstacle, the leveling system demonstrates significant robustness. The implement roll angle deviation is constrained within ±1.5° during both ascending and descending phases, with settling times of 4.05 s and 2.35 s respectively. These results validate the controller’s capability to mitigate sudden terrain-induced disturbances, ensuring operational stability under asymmetric excitation scenarios.

### 3.4. Experimental Analysis

Static experiments were conducted in a controlled environment to validate the fundamental performance of the control system and the effectiveness of the algorithm. Field experiments, conversely, were performed under actual operating conditions to evaluate the system’s practicality and environmental adaptability. Complementing these, terrain mutation tests were designed to specifically assess robustness against sudden disturbances by introducing a 0.15 m step obstacle. 

The comparative results are as follows: static experiments demonstrated that the system could correct the roll angle from ±10° to 0° within 1.3 s, achieving a *MaxAE* of 0.36°. Field experiments showed a *MaxAE* of 0.91° due to environmental complexities. Terrain mutation tests revealed a maximum roll deviation of 1.43° when counteracting a 5° sudden tilt induced by the step obstacle, with settling times of 4.05 s (ascent) and 2.35 s (descent).

These results indicate a controlled precision degradation from static to field conditions, while the mutation test proves the system’s critical ability to constrain deviations within ±1.5° despite extreme asymmetric excitations. The precision discrepancies primarily stem from: (1) vehicle body vibration induced by traversing ground protrusions or depressions, (2) abrupt implement load impacts caused by spatial heterogeneity of field soil, and (3) the asymmetric motion characteristics of the hydraulic cylinder during continuous fine adjustments further amplifying cumulative errors.

## 4. Discussion

This study designed a tractor-mounted implement’s automatic leveling system. Through static and field tests, the system was validated to exhibit high control accuracy, stability, and favorable dynamic response capabilities, meeting the implement’s leveling requirements of tractor-mounted implements during field operations. Comparative experiments demonstrated that the dual closed-loop fuzzy PID algorithm was better suited to the actual control needs of this system than the conventional fuzzy PID algorithm.

Through comprehensive analysis of experimental data and machine kinematic states, it is found that non-ideal motion characteristics of the actuator constitute the primary source of leveling errors. The theoretical model relating machine roll angle to cylinder stroke was originally formulated under the assumption of synchronized piston rod strokes in left and right attitude control cylinders. However, during actual operations, the left and right cylinders exhibit asymmetric motion characteristics due to differences in the effective piston areas on opposing sides of the hydraulic cylinder pistons [23]. This phenomenon shifts the machine’s actual rotation center from its geometric centroid, resulting in a 10.00–13.33% deviation rate between model-predicted and experimentally observed adjustment times per 1° roll angle change [27]. Such discrepancies directly compromise attitude control precision while increasing susceptibility to overshoot phenomena.

The leveling actuator selected for the implement leveling system in this study is a double-acting single-rod hydraulic cylinder, which constitutes an asymmetric hydraulic cylinder with unequal effective areas on both sides of the piston. When the system initiates motion from a stationary state to respond to control commands, the disparity in effective working areas across the piston results in asymmetric initial driving forces and response speeds. Additionally, during actual operation, factors such as hydraulic oil compressibility and pipeline pressure losses influence internal pressure dynamics within the hydraulic cylinder. These collectively cause deviations between the actual piston rod motion and the desired motion during the initial movement phase.

Furthermore, in deriving the relationship model between roll angle and hydraulic control system response time, an averaging method was employed to analyze the response time of the attitude control cylinder relative to piston rod displacement. This simplification facilitated analysis and reduced computational load but excluded the cylinder’s area ratio coefficient from the model. Consequently, during stable operation, systematic predictive discrepancies arise between the actual piston rod displacement and the theoretical model. Even after parameter optimization, the dual closed-loop fuzzy PID controller cannot fully eliminate these deviations during operation. The unmitigated errors progressively accumulate during continuous attitude adjustment cycles, ultimately degrading overall control precision and adjustment efficiency. To enhance system performance, it is recommended that future research incorporates the piston area ratio coefficient of the hydraulic cylinder to modify the existing theoretical model for the relationship between roll angle and piston rod stroke, and to develop an improved model that accounts for asymmetric motion characteristics [31]. This approach could further reduce system operation errors and improve attitude adjustment efficiency.

As the core sensing component of the system, the attitude sensor decisively determines the overall control performance of the leveling system. Consequently, rigorously investigating the impact of environmental factors (e.g., temperature fluctuations, mechanical vibration, electromagnetic interference) on its measurement accuracy is critical for evaluating and enhancing the system’s practical applicability. In subsequent studies, we will conduct a series of controlled environmental tests to quantitatively analyze variations in sensor performance under diverse operating conditions, thereby establishing reliable operational boundaries for the system.

Furthermore, due to field accessibility constraints, only single-run trials were conducted along Paths A → B and $B_{1}\;\to {\;A}_{1}$. While these preliminary results validate the field functionality of the leveling system, future studies should incorporate multiple replicated trials following the planned path sequences. Such replications will enable ANOVA-based quantification of control precision and stability metrics, providing statistically robust performance verification.

## 5. Conclusions

This study presents the design of a tractor-mounted implement’s automatic leveling system based on a dual closed-loop fuzzy PID control algorithm. The system automatically adjusts the implement’s roll attitude in real time according to the preset and actual roll angles. The main findings are summarized as follows.

(1) An automatic leveling system for tractor-mounted implements was designed, which uses an attitude angle sensor to monitor the implement’s roll angle in real time. By employing a dual closed-loop fuzzy PID algorithm, the system regulates the spool position and dwell time of the solenoid directional valve, adjusting the piston rod stroke of the roll attitude control cylinder to achieve precise control over the implement’s roll attitude during operation.

(2) Static test results demonstrated that when the implement’s initial roll angle was ±10°, the system stabilized the roll angle to 0° within 1.30 s, with a *MaxAE* of 0.36° and negligible overshoot. The dual closed-loop fuzzy PID algorithm outperformed the conventional fuzzy PID algorithm in controlling the implement’s leveling, showing superior regulatory accuracy and dynamic response.

(3) Field test results demonstrated that under the dual closed-loop fuzzy PID control algorithm, the leveling exhibited a *MaxAE* of 0.91°, an *RMSE* of 0.28°, and an *MAE* of 0.19°, after implement leveling. The leveling error was controlled within $0.50^{{}^{\circ}}$ for 94.48% of the operational time. 

(4) Terrain mutation tests validated exceptional robustness against sudden disturbances: when traversing a 0.15 m step obstacle inducing 5° roll tilt, the system constrained implement deviation within ±1.5° (*MaxAE* of 1.43°) with recovery times of 4.05 s (ascent) and 2.35 s (descent).

Tests have demonstrated that the designed automatic leveling system for tractor-mounted implements exhibits rapid response speed, high control accuracy, and robust stability, effectively meeting the control requirements for precision agricultural operations.

## Figures and Tables

**Table 1:** Key component models and parameters in hydraulic system.

Name	Model	Principal Technical Specifications	
		Maximum Operating Pressure	Maximum Operating Flow Rate
Three-position four-way solenoid directional valve	4WE10G-3X/CD12	35 MPa	120 L/min
Pressure relief valve	MRV-03P	25 MPa	80 L/min
Tractor hydraulic pump	DDG1A259F9H9-L165	20 MPa	50 L/min

**Table 2:** $\Delta K_{p}$, $\Delta K_{i}$, and $\Delta K_{d}$ fuzzy variable control rules.

$\Delta K_{p}$ $/\Delta K_{i}$ $/\Delta K_{d}$		$E_{c}$						
		NB	NM	NS	ZO	PS	PM	PB
***E***	NB	PB/NB/PS	PB/NB/NS	PM/NM/NB	PM/NM/NB	PS/NS/NB	ZO/ZO/NM	ZO/ZO/PS
	NM	PB/NB/PS	PB/NB/NS	PM/NM/NB	PS/NS/NM	PS/NS/NM	ZO/ZO/NS	NS/ZO/ZO
	NS	PM/NB/ZO	PM/NM/NS	PM/NS/NM	PS/NS/NM	ZO/ZO/NS	NS/PS/NS	NS/PS/ZO
	ZO	PM/NM/ZO	PM/NM/NS	PS/NS/NS	ZO/ZO/NS	NS/PS/NS	NM/PM/NS	NM/PM/ZO
	PS	PS/NM/ZO	PS/NS/ZO	ZO/ZO/ZO	NS/PS/ZO	NS/PS/ZO	NM/PM/ZO	NM/PB/ZO
	PM	PS/ZO/PB	ZO/ZO/NS	NS/PS/PS	NM/PS/NS	NM/PM/PS	NM/PBPS	NB/PB/PB
	PB	ZO/ZO/PB	ZO/ZO/PM	NM/PS/PM	NM/PM/PM	NM/PM/PS	NB/PB/PS	NB/PB/PB

**Table 3:** Main parameters of simulation model.

Parameters	Number of Values
Piston rod diameter of the cylinder	0.05 m
Piston diameter of the cylinder	0.08 m
Load capacity of a single cylinder	1000 kg
Natural frequency of three-way four-port solenoid valve	80 Hz
Damping ratio of three-way four-port solenoid valve	0.8
Steady-state flow gain of three-way four-port solenoid valve	$0.056\;{(m}^{2}\cdot s^{-1})$
Volume modulus of elasticity of hydraulic fluid	${7\;\times \;10}^{8}$ Pa
Hydraulic damping coefficient	0.2
Hydraulic pump operating pressure	18 MPa
Hydraulic pump flow rate	25 L/min

**Table 4:** Field test data analysis.

Planned Driving Path		*Maxae* (°)	$\bar{B}$ (°)	*Mae* (°)	***Rmse* (°)**	Percentage of Leveling Errors Within 0.5° (%)
Dual closed-loop fuzzy PID control algorithm	A→B	0.91	0.10	0.19	0.28	90.56
	$B_{1}\to A_{1}$	0.85	0.04	0.19	0.25	94.48
Fuzzy PID control algorithm	A→B	1.34	0.12	0.24	0.36	88.33
	$B_{1}\to A_{1}$	1.09	0.08	0.32	0.40	76.49

## Data Availability

The original contributions presented in the study are included in the article, further inquiries can be directed to the corresponding author.
